# Inclusive Design in the Field of Education from the Paradigm of Early Intervention

**DOI:** 10.3390/children8060474

**Published:** 2021-06-04

**Authors:** María-Luisa Benítez-Lugo, Elena Pinero-Pinto, Fatima Leon-Larios, Esther María Medrano-Sánchez, Maria de-la-Casa-Almeida, Carmen Suarez-Serrano

**Affiliations:** 1 Department of Physiotherapy, Faculty of Nursing, Physiotherapy and Podiatry, University of Seville, 41009 Seville, Spain; marisabeni@us.es (M.-L.B.-L.); emedrano@us.es (E.M.M.-S.); mcasa@us.es (M.d.-l.-C.-A.); csuarez@us.es (C.S.-S.); 2 Department of Nursing, Faculty of Nursing, Physiotherapy and Podiatry, University of Seville, 41009 Seville, Spain; fatimaleon@us.es

**Keywords:** students, learning activities, game-based learning, creativity, inclusive design, diversity

## Abstract

Inclusive education and early intervention go hand in hand in the early educational stages to reach the maximum potential of the student body. The aim of this study was to analyze the inclusive profile of an educational center and assess the effectiveness of an inclusive task (designed for this study) in a group of students of early childhood education. This analytical, prospective, descriptive and longitudinal study was conducted from both qualitative and quantitative approaches. From the qualitative approach, an interview was carried out with early childhood education teachers. A total of nine participants were interviewed. Their mean age was 42.25 ± 9.30 years, with a mean experience of 14.25 ± 9.25 years. The quantitative part of the study was carried out with 97 students of early childhood education. After delivering a learning workshop about awareness of functional diversity, three variables were analyzed: story memory, demonstrated emotion, and game memory. The qualitative study indicates that it is necessary to develop coping strategies, such as including special education tasks in the classroom, prior to specific staff training and programming in specific aspects of awareness. Moreover, it is shown that the perception of treatment among peers is already present at this educational stage. The quantitative study reveals that the task was exciting and motivating for the students, which promotes learning and awareness.

## 1. Introduction

The number of people with functional diversity under 6 years of age is increasing considerably. Around 2.5 million people in Spain have a degree of disability who need special attention, of which 108,570 are minors [1]. The respective interventions available to people with functional diversity have evolved from total exclusion from educational and social services to their current state, where the Spanish Educational legislation supports full inclusion in the framework of an education for everyone. In this context, the concept of inclusive education establishes that all educational centers must be able to accommodate all students. In this sense, the aim is to achieve a quality education in which inclusive schools provide children with the opportunity to interact with each other and expand their learning, since this context values and respects differences [2]; therefore, it is a strengthening process to reach all students, focusing the interventions on the classroom and developing the teaching-learning process in a collaborative manner. At the international level, several authors support inclusion as an effective idea through which the teaching, learning, achievements, attitudes, and wellbeing of each student is important [3], providing different scenarios and new opportunities for people who may have educational difficulties. In this line, other authors consider that an inclusive school in early childhood education, with effective leadership, allows children to feel capable of developing the basic skills for life [4].

Furthermore, we frame early intervention within the educational period of the first and second cycles. Although early intervention comprises three essential spheres, greater attention is paid to those interventions focused on the child and the family, disregarding those interventions focused on the environment. Thus, it is necessary to develop strategies within the educational scope, with the double aim of framing the interventions within the environment and ensuring the inclusion of children with functional diversity or at risk of disorder.

Studies about educational inclusion can be classified into three research lines [5]:

- Inclusion and its effects on students, teachers, and families.
- Research on teaching in inclusive classrooms.
- Beliefs, perceptions, and attitudes toward inclusion.

A fourth research line is the voice of students [6], who are the real protagonists.

In addition to these clarifications, it is important to highlight that the European Agency for Special Needs and Inclusive Education [7], which is an independent and self-governing organization, proposed the attainment of an inclusive school for everyone. It is time to analyze the state of such a pursuit and support actions that favour it. It is important to evaluate all the information that may be considered or collected to achieve effective inclusion, as well as sensitivity and awareness of functional diversity.

In this sense, considering the relevance of teacher training in inclusion and all the advantages of developing inclusive activities for all students, the aim of the present study was to analyze the inclusive profile of an educational center in the city of Seville, as well as to assess the efficacy of an inclusive task (designed for this study) in a group of students of early childhood education.

## 2. Materials and Methods

This is an analytical, prospective, descriptive, and longitudinal study conducted from both qualitative and quantitative approaches, which were focused on the teachers and students, respectively. We created a list of educational centers in the city of Seville that were close to areas of different cultural and socioeconomic level. The management teams of these educational centers were invited via e-mail to participate in this study. Of all the invitations, one center located in the southern district agreed to participate. After a meeting with the board of directors and the families of the center, we received their informed consent. Written informed consent was obtained after explaining the study. The Institutional Review Board of the University Hospital Virgen Macarena of the University of Seville approved this research. (15 December 2019).

### 2.1. Characteristics of the Sample and Variables

In the first phase of the study, a semi-structured interview was conducted with the teachers of the second cycle of early childhood education. This educational cycle was selected to frame the study within the paradigm of early intervention, establishing an intervention from the environment. The inclusion criteria established that the students had to be in the second cycle of early childhood education, i.e., aged 3–6 years, and have the informed consent of their parents. On the other hand, the study excluded students who missed the day in which the creative task was carried out in the classroom, as well as those who had joined the center in the academic course in which the study was conducted.

To respond to the question “what activities are carried out in the educational center?”, a structured interview was designed for the qualitative part of the study (Table 1).

For the qualitative part of the study, which was conducted with the teachers of the educational center, a digital recorder was used (Olympus VN-5500PC, Olympus, Tokio, Japan). In addition to this, the significant sentences said during the interviews were written down in a diagram with the structured questions, highlighting those related to training in functional diversity, inclusion in the classroom or the benefits of intervening in children with special educational needs.

For the intervention with the students, a creative task was designed to raise awareness and sensitize them to functional diversity. This task consisted of the telling and interpretation of the tale of “Carlota the Turtle” and participation in a game in which the students had to overcome a challenge simulating a physical, cognitive, sensory, or speech disability. In this study, we used an extensive strategy, applying a questionnaire six months after the creative task was carried out. Table 2 shows the dimensions analyzed and the indices included in the questionnaire administered to the students.

Both lines of work include the obtained results in a matrix with coded indices and the data of all participants, which were guarded by the principal investigator, following Law 15/1999 on Personal Data Protection.

### 2.2. Development of the Creative Task “Awareness and Sensitization to Functional Diversity”

**Figure 1:**
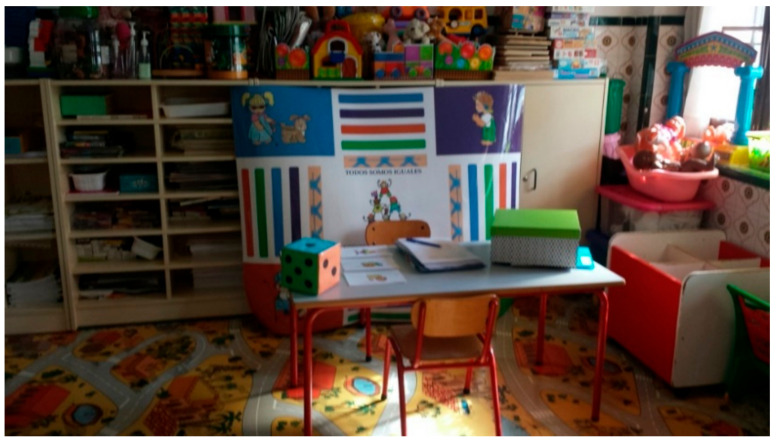
Materials and props of the creative workshop.

The designed task can be framed as an ordinary measure to attend to the needs of students in the classroom [8], under the label of a learning workshop. The task was carried out in small groups, focusing on a specific topic, which, in this case, was awareness and sensitization to functional diversity, developing different tasks that concluded with an activity: solving a puzzle as a group. This task was performed in groups, with a maximum of 25 students per group, and required a computer and a projector to present the digitalized tale of “Carlota the Turtle”, as well as a game board with different materials (masks, pins, balls, sterognostic bag, pictograms, name of all the participating children in sign language, sound puzzle, pieces of the final puzzle, big die, and a box with the characters of the tale and the different props). Figure 1 shows the designed materials.

### 2.3. Statistical Analysis, Data Organization, and Triangulation of the Methodological Strategies Used

The data obtained from the intervention conducted with the teachers were analyzed using the ATLAS-TI, version 7.5 (Scientific Software development GmbH, Berlin, Germany) whereas those obtained from the intervention conducted with the students were organized in an independent matrix, using SPSS v18.0 statistical software for Windows (SPSS, Chicago, IL, USA). All statistical tests were carried out considering a confidence interval of 95% (*p* < 0.05). For the triangulation of the data, we used the results obtained with the qualitative methodology.

## 3. Results

**Figure 2:**
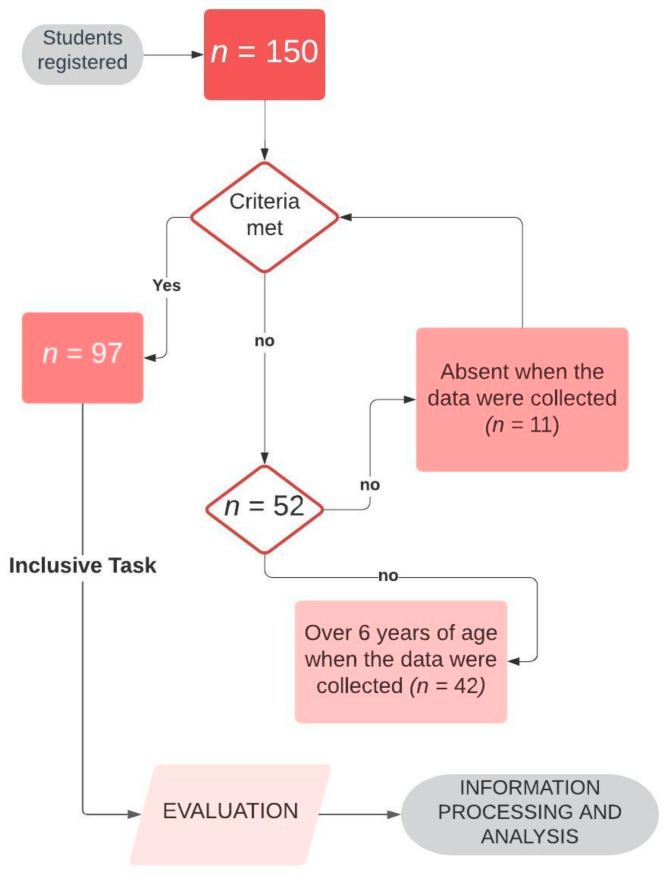
Flowchart of the selection of participants and the creative activity carried out.

The average age of the nine teachers who made up the team of early childhood educators was 42.25 ± 9.30 years, with an average experience of 14.25 ± 9.25 years. The second phase of this design was focused on the students registered in the second cycle of early childhood education, whose parents signed the informed consent, obtaining a final sample of 97 students with an average age of 4.85 ± 0.768 years, a sex proportion of 50.5% boys and 49.5% girls, and the recognition of special educational need in 6.2% of the sample, according to the educational guidance team. Figure 2 displays the flowchart of the selection of students of the second cycle of early childhood education and the creative activity carried out.

### 3.1. Findings of the Qualitative Study: Study on Teachers

Once the textual interviews were loaded into ATLAS-TI, four analytical categories were obtained, based on the questions asked in the semi-structured interview, that can be found in Supplementary Annex S1:

- Coping strategies: existing, desirable.
- Previous teacher training and importance.
- Sensitizsation: opinion and repercussion.
- Perception of the treatment among peers and justification.

### 3.2. Findings of the Quantitative Study: Study in Children

Table 3 shows the descriptors obtained in the variables analyzed through the creative task.

Regarding the degree of memory, 60.8% of the students had a good memory of the tale and of the activity, whereas 27.3% had an acceptable memory and 15.5% had a slight memory. With respect to the emotion expressed when the box with the characters of the tale was uncovered, 93.8% of the students showed empathy, making gestures to take the props and interpret the actions of the tale, and only 6.2% of the sample showed no reaction. With regard to the memory of the game that was played after the tale, 59.8% of the sample described the activities that involved simulating visual, auditory, motor, or intellectual diversity, 11.4% could recall up to the specific task that they got from the die, and only 28.8% of the students had a slight memory.

Regarding the degree of satisfaction with the activity, 88.7% of the students scored 10 points, and only 1% scored less than 5 points (no satisfaction). Table 4 shows the descriptors related to these variables. It is important to highlight that 100% of the students agreed to participate again if the activity was ever repeated.

According to the reviewed literature [9], it is considered important to analyze the possible differences between sexes in the degree of memory of both the tale and the subsequent game. The obtained results show that the level of memory of the tale in the girls was significantly higher with respect to the boys (χ^2^ = 6.39; *p* = 0.041). On the other hand, the degree of memory of the game did not show differences between sexes (χ^2^ = 0.75; *p* = 0.43). The last objective of this study was to analyze whether the respective variables could be correlated with age, using Pearson’s correlation test. The degree of memory of the tale obtained a value of *r* = 0.340 (*p* = 0.001), indicating that the degree of memory increases with age. On the other hand, the degree of memory of the game seems to decrease with the increasing age of the students, showing a significant correlation (*r* = 0.239; *p* = 0.05). Lastly, we analyzed the possible age-related differences in the emotion demonstrated after discovering the characters in the tale. In this case, we obtained a value of *r* = 0.228 (*p* = 0.05), suggesting that emotional indifference decreases with increasing age.

## 4. Discussion

### 4.1. Inclusive Activities Performed in the Educational Centre

In the “coping strategies” category, the first activity highlighted by the teachers of this center was the adaptation of activities for children with educational needs within the classroom, using the inclusive classroom only in cases of very specific needs. This measure allows normalizing the characteristics of each child, with innocence and the intervention of the tutor in the classroom being the most important support elements. This priority for intervening in the classroom is in line with the document published by the specific agency for special needs [10] and other studies [11], which state that children learn better through daily experiences and by interacting with their usual caregivers in day-to-day contexts, understanding that, in the educational center, the best strategy involves the classroom and their classmates. In this case, the benefits of working in natural environments are pointed out [12].

Other authors have drawn the same conclusions, including the need to remove the support in learning when it is no longer required [9]. In this sense, this center works within the classroom, although it does not include individualized work through routines, which is the best intervention for an inclusive approach. The lack of time and resources could be the cause of such routines. In this category, it is highlighted that, although the programs of the teachers did not specifically include awareness and sensitization activities in matters of functional diversity, they do have other activities that, in a transversal manner, promote values required to empathize with diversity.

Regarding the importance of teacher training in carrying out inclusive strategies, these teachers expressed the same concerns and worries as those found in the international studies conducted by the European Agency for the Development of Special Needs and Inclusive Education. Currently, teacher training in matters of functional diversity is not contemplated; however, it is agreed that teachers need continuous training in order to act with creativity and inclusion, which is in line with the document of the Agency for the Development of Special Education [13,14]. In turn, these data reproduce the studies of other authors [13], who conclude that inclusive classrooms are those in which the teachers have greater training in specific matters of special education or similar [9]. The activities pointed out by the teachers enable a diverse evaluation, provide continuous support, and allow working among peers, promoting the personal and professional growth of all the parties involved in the teaching-learning process [4]. A recent study [15] performed in Thailand highlights the positive experience of teachers with inclusive practices, although it also gathers barriers such as work load, time and the lack of resources.

With respect to the activities of awareness and sensitization in matters of functional diversity, the participants argued that these types of tasks have a benefit that can be extrapolated to the whole of society. In Early Childhood Education, it is agreed that attention must prevent and compensate for all those circumstances that influenced the appearance and development of certain difficulties, understanding that raising awareness about functional diversity can be included in this premise [16]. Regarding the treatment among peers, the interviews showed that children without functional diversity welcome those with specific needs. The innate goodness of children, together with the education in values given by the family and strengthened in the school, could be the cause of this behavior. This finding supports the assertion found in several studies [17,18,19] and in the work carried out by the European Agency for the Development of Special Education, which maintains that inclusive schools benefit everyone. Similarly, it is stated that the success of inclusive actions could be related to the establishment of alliances between sectors that participate in inclusion [20]. This datum reinforces the consolidation of possible agreements between educational centers and other external sectors that may support the needs present in the classroom. However, the degree of inclusion decreases with the increasing degree of diversity recognized by the students [21]. Likewise, other studies state that the contexts may act as both facilitators and barriers [22]. In this sense, the behavior of the students could be explained by the benefits of this type of methodology, such as in the social relationships between peers [23], as well as by all the factors that could be related to the application of tasks. Thus, leadership in students and teachers is highlighted as a key element [24]. In this line, teachers should contribute as leaders to the transformation of the classroom and the family to change the negative attitudes that the community may have toward functional diversity; therefore, the application of activities similar to the one presented in this study can be included among the instruments to be implemented in the classroom [25].

### 4.2. The Creative Task Designed and Carried Out in the Educational Centre

The satisfaction and score obtained in the activity conducted in this study was excellent, showing that it was a motivating task for the students and that both motivation and emotion are the true drivers of any type of learning [26]. In fact, in the educational scope, motivation is understood as an internal process that activates, directs, and maintains behavior toward a specific goal, in which biological, psychological, personality, social, and cognitive variables participate. The child brain is motivated by everything that is perceived as new [27]. In this sense, it is understood that a different task from those programmed in the contents of the course could have activated the neural structures that trigger motivation, especially the right prefrontal cortex, which is related to intrinsic motivation and ethics learning [28]. It seems that what students feel is as important as what they learn. This strengthens learning through active listening, discussion, and the development of empathy and non-verbal communication [29]. Activities that involve playing and achieving goals are based on these pillars and are essential for children [30].

With regard to the learning obtained after carrying out the task, the degree of memory of both the tale and the game was good, as well as the positive emotion demonstrated by the discovery of the characters, despite the young age of the students and the fact that the activity was only conducted once. Although similar experiences have been documented, the time and frequency vary with respect to our activity [31]. In this sense, we believe that the involvement of motivation and emotion in the task could have been fundamental, as it activated the connections between the amygdalin system, the prefrontal cortex, and the hippocampus, which mediate the mnesic processes. Thus, it is stated that childhood is a period of special intensity for declarative memory, involving a strategic process that includes the making of memories, their optimal production, and benefiting from them [8]. This implementation is not always stable and depends on the impact of the task; therefore, sensory-motor experiences are supported as promoters of learning in the age range of 4–7 years, when the dorsolateral cortex is activated in the face of new tasks and attention is enhanced when accompanied by emotional content. These authors identified other elements that could influence learning, such as parenting styles, socioeconomic level, bilingualism, and cultural contexts [32]. These variables become moderators for future research lines. Another finding to explain these results is the involvement of mirror neurons, which allow our brains to correlate our own actions with those of others and make sense of them. Mirror neurons understand the intentions, feelings and emotions of other people [32]. In this sense, we can support the idea that the continuous activation of these neurons throughout the whole execution of the creative task, regardless of whether or not the child was performing the action, could have consolidated the learning intended in the task and enabled the child to empathize in terms of functional diversity. However, it was observed that the degree of memory of the tale was greater in girls than in boys, whereas no differences were found in the memory of the game between sexes. Playing involves spatial activities, and several studies have specified that spatial memory is greater in boys than in girls, with a clear difference in this skill from the age of five years [33]. This fact could explain why, given the average age of the sample, there were no significant differences between the sexes. Regarding the degree of memory, similar findings show that girls obtain better results than boys in tasks that involve verbal memory and learning, immediate memory, and the recovery and understanding of stories through questions and information processing speed [32].

Regarding age, several findings corroborate that learning increases with age, as is shown in a study that analyzed the correlation between episodic memory and the volume of certain structures that make up the hippocampus, showing differences in children from the age of 6 years with respect to those of 4 years of age [32]. Moreover, it is known that working memory, short-term memory, selective attention, and processing speed increase with age [34], all of which are activated in the task performed in the present study.

In this study, the opinions of the teachers confirm that teaching projects do not include tasks that specifically act on the awareness about functional diversity, although there may be others, such as playing and cooperative learning activities, which could promote inclusion. Furthermore, continuous teacher training, the involvement of families, the values provided by the educational center, time, and resources seem to be fundamental elements for inclusive education.

Likewise, the creative task designed to raise awareness and sensitize children to matters of functional diversity was positively valued by the students and met the objectives set by the research team. The learning of this task improved with the increasing age of the children, with the girls consolidating the contents of the story better than the boys and finding no differences between sexes in the rest of the analyzed variables. As future lines of our research, it´s proposed that we carry out a multicenter study, as well as design a program that combines the optimization of development in children with disabilities and emotional education, and to verify its effectiveness and impact on inclusion.

## 5. Conclusions

Training in functional diversity and the development of creative activities are necessary to optimize inclusion. The use of play and emotions as a basis for creative tasks optimizes learning, awareness, empathy, and inclusion in children with and without disabilities in this educational center.

## Figures and Tables

**Table 1:** Dimensions, indicators, and indices of the semi- structured interview with the teachers (qualitative study).

Dimension	Indicator	Index
Experience with children with Special Educational Needs	Number of tutored children who presented any disability	Percentage of children intervened in the professional career of the teachers
Strategies carried out for the sake of inclusion in the classroom	Number of strategies carried out in the classroom	Number of interventions conducted per child and their success
Degree of teacher training in matters of inclusion and disability	Number of courses studied in matters of disability	Valuation of training courses and their relationship with intervention in the classroom
Contents of inclusion in the teaching program of early childhood education	Number of teaching activities carried out	Number of activities conducted in matters of inclusion
Behavior of the children toward classmates with SEN	Number of positive attitudes toward children with SEN	Degree of empathy of the children toward other children with SEN and explanation of this behavior
Degree of importance of awareness and sensitization activities in matters of disability	Number of potential advantages of this type of activities for the classroom, the families and society	Percentage of positive responses towards the importance of including these types of activities

**Table 2:** Dimensions, indicators, and indices of the questionnaire focused on the children (quantitative).

Dimension	Indicator	Index
Degree of memory of the tale used for the task	Number of key descriptors of the tale: - Turtle - White or colorless - She could not hide - She was always found - Friends - She was covered with flowers - She was covered with butterflies - She was angry because she could not play - She was sad - She could hide in the snow - Everyone could play	Number of descriptors mentioned by the child when asked about the tale of Carlota the Turtle. From 0 to 3 points → slight memory From 4 to 6 points → acceptable memory From 7 to 9 points → Very good memory From 10 to 11 points → Optimal memory
Degree of emotion demonstrated with the characters of the tale in three dimensions	Expression shown when uncovering the box with the characters of the tale and the props of the story	The facial expression of the student is evaluated when the box is uncovered: If the child smiles and seems attentive, even trying to play with the characters → Optimal empathy (2 points) If the expression of the child does not change → 1 point If the child is angry or disgusted → Low empathy (0 points)
Degree of memory of the board-and-dice game	Number of key descriptors of the game used: - Dice - Colorful board - Eyes closed - No speaking - Feet or arms together or any other difficulty - Pictograms - Final puzzle	The number of descriptors mentioned by the child is scored. From 0 to 2 points → slight memory From 3 to 5 points → Acceptable memory More than 5 points → optimal memory
Score of student satisfaction with the creative task	The students were asked to state, from 0 to 10 on a 10 cm tape, the degree of satisfaction they felt with the activity	Based on the student satisfaction: From 0 to 4.99 points → Unsatisfied with the task From 5 to 6.99 → Good satisfaction From 7 to 8.99→ Considerable satisfaction From 9 to 10→ Optimal satisfaction

**Table 3:** Descriptors obtained in the variable analyzed.

Variables	Mean (Maximum-Minimum)	Standard Deviation
Degree of memory of the tale	7.04 (12-0)	2.937
Degree of emotion demonstrated	1.94 (2-1)	0.242
Degree of memory of the game	3.45 (7-0)	1.581

**Table 4:** Satisfaction with the activity.

Variables	Mean (Maximum-Minimum)	Standard Deviation
Degree of satisfaction	1 (1-1)	0.00
Would participate again	1 (1-1)	0.00
Score of the activity	9.74 (10-3)	0.916004

## Data Availability

The data that support the reported results are guarded by the principal researcher María-Luisa Benítez-Lugo (marisabeni@us.es).

## Supplementary Materials

The following are available online at https://www.mdpi.com/article/10.3390/children8060474/s1. Annex S1—Semi-Structured interview.
