# Supplementary material for: Inclusive Design in the Field of Education from the Paradigm of Early Intervention
Source: Children (Basel). 2021 Jun 4;8(6):474. doi: 10.3390/children8060474 (PMC8228456; doi:10.3390/children8060474)
Supplement: Supplementary file 1 [file children-08-00474-s001.zip › children-1231113-supplementary.pdf]

SEMI-STRUCTURED INTERVIEW WITH EARLY CHILDHOOD EDUCATION TEACHERS

Teacher data:
Years of teaching: Age:

- Approximately how many children with Special Educational Needs have you had in the classroom throughout your professional career? What disability did they present in general?

- What strategies have been carried out for their integration in the classroom?

- Have you received any specific training in relation to this topic? From 1 to 10, what would your score be?

0	1	2	3	4	5	6	7	8	9	10

- Does the teaching program include any group activities to promote integration?

- Among these possible activities ... is there any specific one that works on disability awareness?

- In your professional career, how have you perceived the behavior of the children in the classroom towards a possible child with Special Educational Needs?

- How would you justify this possible behavior?

- Do you consider it interesting to include awareness-raising activities in a mandatory way in all children's studies/curricula?

- What do you think this type of activity could affect or achieve?
